# Mass production of a S-layer protein of *Bacillus thuringiensis* and its toxicity to the cattle tick *Rhipicephalus microplus*

**DOI:** 10.1038/s41598-019-53854-3

**Published:** 2019-11-26

**Authors:** Caleb C. Lormendez, Manuel Fernandez-Ruvalcaba, Markis Adames-Mancebo, Victor Manuel Hernandez-Velazquez, Fernando Zuñiga-Navarrete, Gabriela Flores-Ramirez, Laura Lina-Garcia, Guadalupe Peña-Chora

**Affiliations:** 1 Universidad Autonoma del Estado de Morelos, Centro de Investigacion en Biotecnologia, Av. Universidad 1001, Colonia Chamilpa, CP 62209 Cuernavaca, Morelos Mexico; 2 Centro Nacional de Investigaciones en Parasitologia Veterinaria INIFAP, Km. 11.5 Carretera Federal Cuernavaca, Cuautla, Col. Progreso, Jiutepec, Morelos CP 62550 Mexico; 3 Institute of Virology, Biomedical Research Center, Slovak Academy of Sciences, Dúbravská Cesta 9, 845 05 Bratislava, Slovakia; 4 Universidad Autonoma del Estado de Morelos, Centro de Investigaciones Biologicas, Avenida Universidad 1001, Colonia Chamilpa, CP 62209 Cuernavaca, Morelos Mexico

**Keywords:** Industrial microbiology, Agroecology

## Abstract

The most commonly used biopesticides to control agricultural, forest and insect vectors of human diseases are derived from the bacterium *Bacillus thuringiensis*, which begins to produce Cry and Cyt insecticidal proteins during the onset of the sporulation phase. Some *B*. *thuringiensis* strains also produce S-layer proteins that are toxic to certain pests. S-layer proteins are the most abundant proteins in bacteria and archaea. This proteins’ key trait to design high performace processes for mass production is their continuous expression during the vegetative phase, unlike Cry and Cyt, which are restricted to the sporulation phase. In this work, a S-layer protein expressed by the GP543 strain of *B*. *thuringiensis* that is toxic to the cattle tick *Rhipicephalus microplus* was mass produced using the batch culture fermentation technique. In addition, the spore-protein complex showed a mortality rate of 75% with a dose of 300 µg·mL^−1^ on adult females of *R*. *microplus* after fourteen days. The lethal concentration 50 was 69.7 µg·mL^−1^. The treatment also caused a decrease of 13% in the weight of the mass of oviposited eggs with 200 µg·mL^−1^ of the spore-protein complex and inhibition of the hatching of eggs from 80 to 92%. Therefore, this could be a good option for controlling this parasite. The advantages of S-layer protein synthesis are focused on the production of a new generation of proteins in pest control. This is the first report on the mass production of an S-layer protein that is responsible for toxicity.

## Introduction

*B*. *thuringiensis* has 57% of the world’s biopesticide market, and it is by far the best alternative to chemical insecticides for pest control^1^ with the advantage of being safe to non-target organisms. The production of *B*. *thuringiensis* in bioreactors has been performed just with a few strains that synthesize δ-endotoxins, which comprise two family proteins, Cry and Cyt, that crystallize in parasporal bodies and have widespread activity against insect pests. However, in the majority of the strains, protein synthesis is related to the sporulation phase^2^. Additionally, spores and Cry protein yields are usually low^3^, so they often cannot compete with chemical insecticides. Furthermore, some strains express other protoxins in the vegetative phase (VIP, β-exotoxins and S-layer), and some virulence factors have synergism with the Cry proteins, thereby increasing toxicity to its host^4^.

S-layer proteins are most abundant in archaea and bacteria; the first report demonstrating that a S-layer protein was involved in toxicity to an insect pest was against *Epilachna varivestis*^5^, and this S-layer protein represented a new group of proteins that could be used in insect pest control. The importance of this kind of protein is that it is expressed in the vegetative phase. This could have favourable implications in mass production, such as the design of a fed-batch process and the modification of feeding rates of carbon sources to overcome catabolite repression and enhance the production of the S-layer protein^6^ without using modified organisms. This research was focused on the production of the S-layer protein by batch fermentation with the strain *B*. *thuringiensis* GP543 that synthesizes it in parasporal crystalline inclusions; this protein has toxic activity *in vitro* and *in vivo* against adult females of the cattle tick *Rhipicephalus microplus*^7^.

*R*. *microplus* is the most harmful ectoparasite affecting the livestock industry and is widely distributed in tropical and subtropical areas^8^. This tick is a vector of protozoa, spirochaetes, rickettsiae and viruses that cause diseases in livestock, humans and companion animals. It is controlled by chemical acaricides, but it has developed resistance^9^, and some strains are multiresistant^10–12^. Therefore, it is of great interest to develop a bioacaricide for the control of this pest, which has lost sensitivity to chemical products. In concordance, the S-layer protein produced by the strain GP543 of *B*. *thuringiensis* has shown toxic activity to *R*. *microplus* and could be an option for chemical acaricides.

## Results

### Identification of the S-layer protein from the GP543 *Bacillus thuringiensis* strain

**Figure 1:**
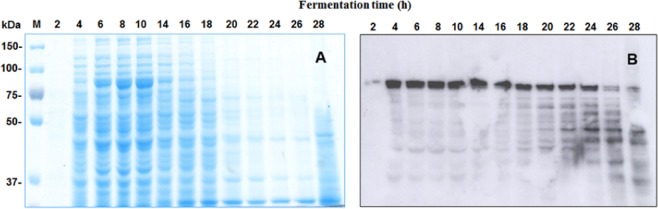
Protein profile synthesized during kinetic growth of the GP543 strain grown in GYS medium. Samples of culture from a batch fermentation were taken every two hours until 28 hours of culture was reached. The samples were resolved by SDS-PAGE in duplicate: (**A**) protein profile in SDS-PAGE gel stained with Coomassie blue (**B**) Western blot analysis using a specific anti-S-layer antibody detected the production of the S-layer protein (around 100 kDa).

The protein profile was analysed with respect to the kinetics of bacterial growth using SDS-PAGE gels in denaturing conditions. Samples from every two hours of fermentation were run in duplicate (Fig. 1A) and one gel was stained with Coomassie blue while the other (Fig. 1B) was transferred to PVDF membrane for western blot detection. The protein of interest (close to 100 kDa, GP543-SL) was observed at the first hours of vegetative growth, reaching a maximum at hour 10 coinciding with the beginning of the sporulation and crystalline inclusions formation processes. The band of around 100 kDa was analysed by MALDI-TOF mass spectrometer and the MASCOT search results indicated that the peptide mass fingerprinting (PMF) of the sequenced protein is 58% similar to a S-layer protein from *Bacillus thuringiensis* (AAY28601.1) (Suplemental 1). This band was also the predominantly detected in the Western blot assay performed with a S-layer antibody (Fig. 1B).

After 14 hours of culture, the 100 kDa protein began to degrade and the degradation products coincided with the detection of several bands of low molecular weight in the Western blot (Fig. 1B). In addition, this result confirms that the protein is continuously expressed until the exponential phase and decreases in concentration at the end of the stationary phase.

### Batch fermentation

**Figure 2:**
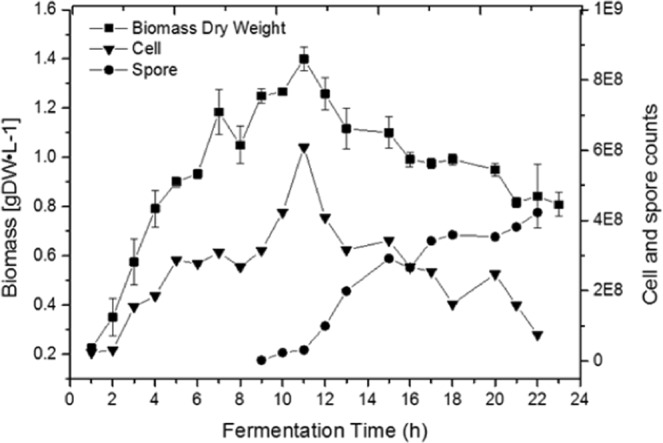
Kinetics of growth in dry weight and the number of vegetative cells and spores of the GP543 strain in GYS culture medium in a stirred tank bioreactor. Data are represented as the mean value ± SE.

S-layer proteins are continuously expressed, which allows high-performance processes such as fed-batch culture; therefore, it is necessary to know the values associated to cell duplication (Luedeking-Piret equation). To this end, the values of cell growth, sporulation and production were recorded every hour in GYS medium that contained 10 g · L^−1^ of glucose. The conversion to biomass and product was analysed as well as whether this conversion was related to the carbon source in a process of controlled feeding for biomass production and S-layer protein synthesis. Figure 2 shows that during the first hours, the GP543 strain exhibited exponential growth until eleven hours with µ = 0.42 h^−1^, R² = 0.94 and Xmax = 1.4 gDW · L^−1^, Y_S/X_ = 0.14, resulting in an increase in the concentration of vegetative cells reaching a value of 6 × 10^8^ after 11 h of growth. In the second growth phase, a rapid decrease in vegetative cell concentration but an increase in spore formation was observed. Additionally, we observed the presence of spores after nine hours of culture and continued until more than 95% of sporulation was reached at twenty-three hours.

**Figure 3:**
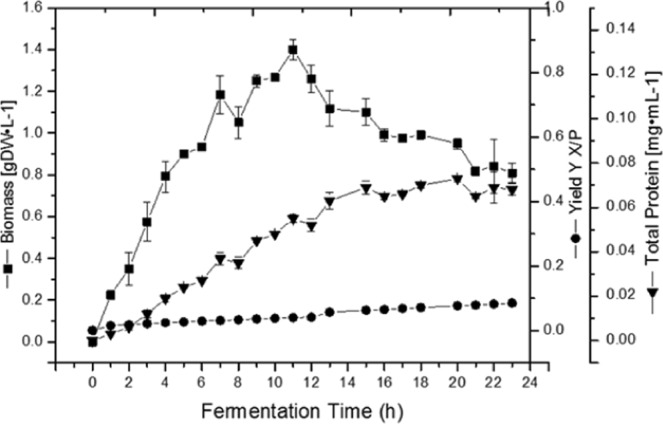
Kinetics of the growth in dry weight, total protein and yield Y_X/P_ of the GP543 strain in GYS culture medium in a stirred tank bioreactor. Data are represented as the mean value ± SE.

The relationship between the protein production as a function of the stage of the culture and the biomass concentration was analysed by yield (Y_X/P_), productivity (Qp) and the Luedeking-Piret equation, and the production and yield are shown in Fig. 3.

**Figure 4:**
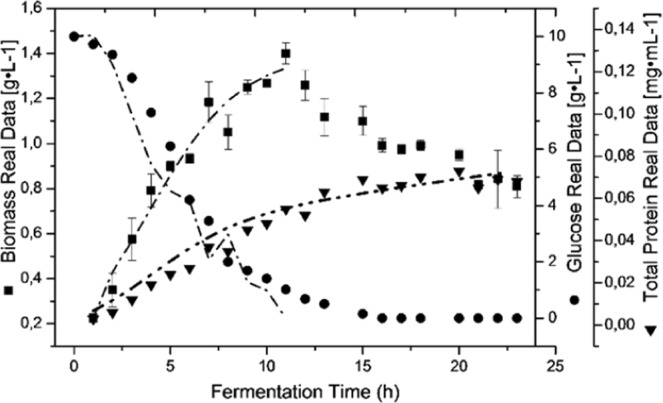
Comparison between the kinetic simulated using the calculated model and the kinetic obtained from experimental data for the GP543 strain cultured in GYS medium in a stirred tank bioreactor. Data are represented as the mean value ± SE.

Regarding the protein production, which increased until eighteen hours and then remained stable until the end of the culture, the maximum protein concentration was P = 0.07 mg · mL^−1^ and even when the culture entered sporulation at eleven hours, the synthesis of protein continued during four hours more. The biomass production kept a steady exponential growth until the twelve hours, when a slight decline that was maintained until the end of the culture started. When solving the equations of the logistic growth model applied to the fermentation batch, the growth parameters can be calculated and predicted with precision, including the constants of associated and non-associated cell growth, as shown in Fig. 4. The symbols are the real data of biomass, protein and glucose consumption, and the solid lines are the modelling. The model reliably represents each of these parameters and allows calculation of the constants α = 0.033 and β = 0.001 with R^2^ = 0.98 for every parameter. It was also observed that the 1.4 gL^−1^ of glucose added when the culture entered sporulation at eleven hours was consumed after sixteen hours of fermentation.

### Immersion bioassay with spore protein S-layer complex

**Figure 5:**
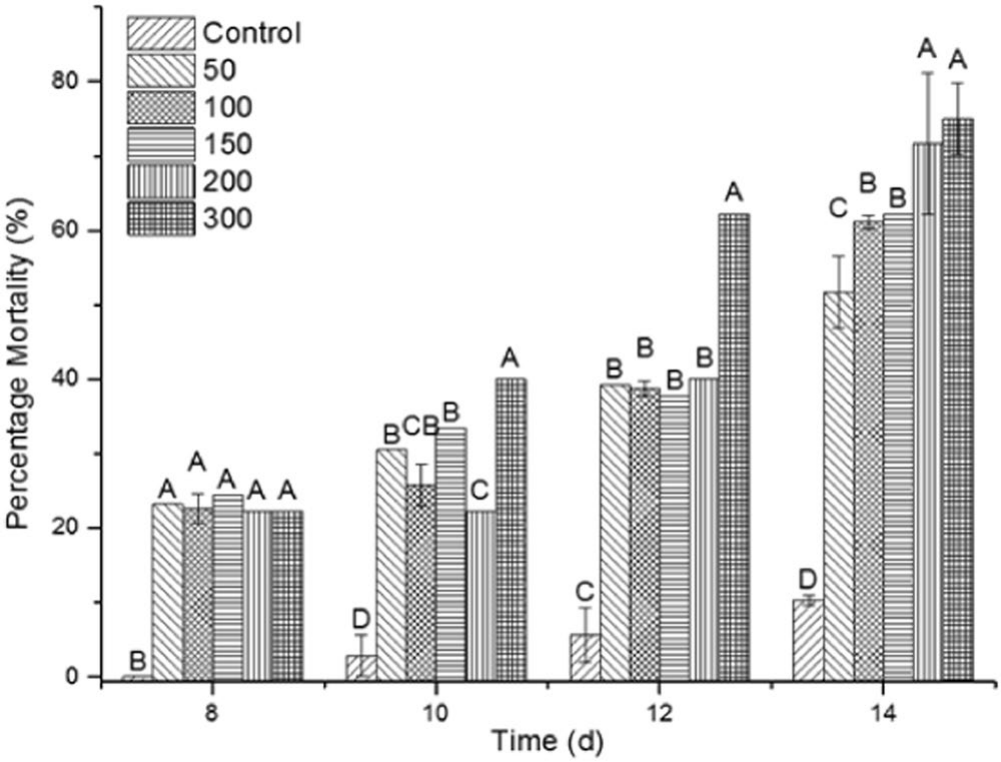
Virulence of the spore-protein S-layer complex of the *B*. *thuringiensis* strain GP543 against *R*. *microplus*. According to Tukey’s test, values in each column of each treatment with the different letter differ significantly. Data are represented as the mean value ± SE.

The spore-protein S-layer complex of the GP543 strain of *B*. *thuringiensis* was produced in bioreactor and its virulence against *R*. *microplus* tested by immersion bioassays. Seven hundred engorged thicks were homogenized by weight and separated into experimental units. Different concentrations of spore-protein complex (0, 50, 100, 150, 200 and 300 µg · mL^−1^) were tested against the ticks and the mortality rates were measured in each case. As shown in Fig. 5, the lowest concentration of the complex caused more than 50.0% mortality at fourteen days, and the highest mortality was 75.0% with 300 µg · mL^−1^ on this day. The statistical analysis shows that at eight days, there were no significant differences between treatments, however, after twelve days of treatment, the concentration of 300 µg · mL^−1^ caused a statistically significant higher mortality. Under this conditions, the spore-S-layer complex showed a 50% lethal concentration (LC 50) of 69.7 µg · mL^−1^.

**Figure 6:**
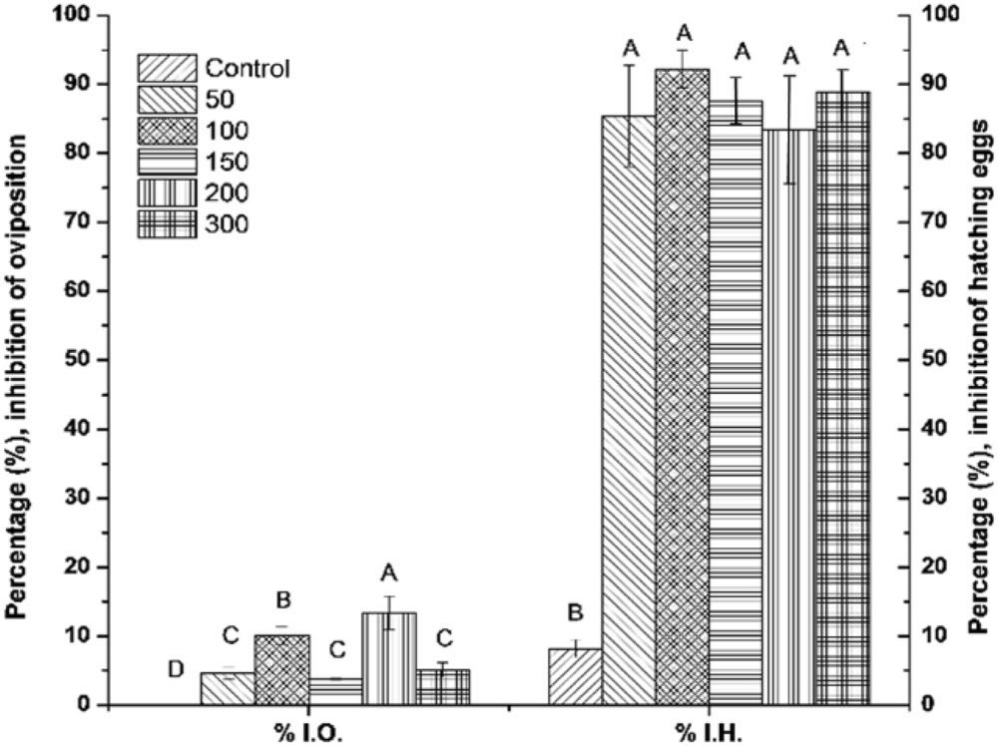
Inhibition of oviposition (% I.O.) and the hatching of eggs (% I.H.) caused by the spore-S-layer protein complex of the *B*. *thuringiensis* strain GP543.

Interestingly, several ticks that survived the treatments with the spore-S-layer complex were unable to ovoposite (% I.O.) or laid eggs masses with decreased weight. The greatest inhibition of oviposition was caused by the 200 µg · mL^−1^ concentration, which caused an inhibition of 13.4%, as shown in Fig. 6. However, not only the oviposition was affected but also the viability (% I. H.), with the highest inhibition of the hatching of eggs at the 100 µg · mL^−1^ concentration. At the end of the inhibition assay, all doses tested showed greater than 85% inhibition of the hatching.

### Immersion bioassay with purified S-layer protein

**Figure 7:**
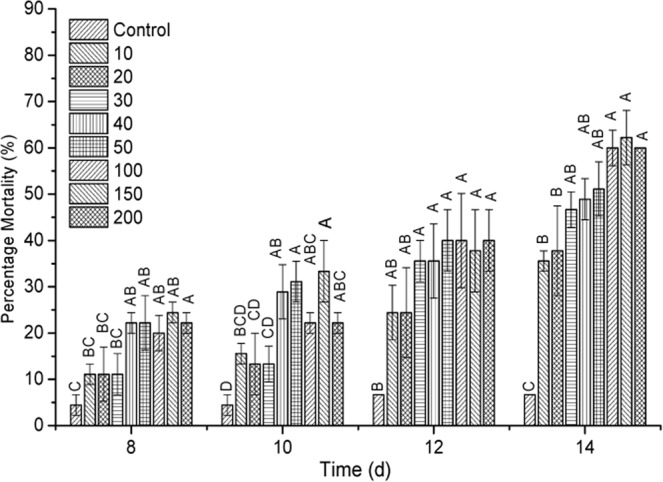
Virulence of purified S-layer protein of the *B*. *thuringiensis* strain GP543 against *R*. *microplus*. Measures in each column of each treatment with the different letter differ significantly, accordingly to Tukey’s test. Data are represented as the mean value ± SE.

To test the virulence of the purified S-layer protein, the engorged ticks were exposed to several concentrations of purified S-layer protein (0, 10, 20, 30, 40, 50, 100, 150 and 200 µg · mL^−1^). As shown in Fig. 7, the S-layer protein was toxic by itself to the ticks since all the tested concentrations are statistically different to the control. The lowest concentrations of 10, 20 and 30 µg · mL^−1^ showed a very similar mortality of less than 46.67%, with no significant statistical differences throughout the bioassay. In a similar way, the higher concentrations of 100, 150, 200 µg · mL^−1^ showed a similar effect among themselves, causing mortalities between 60 and 62.22% (Fig. 7) at 14 days. However, the two groups of concentrations (low and high) were statistically different. The pure protein showed a LC_50_ of 75.72 µg · mL^−1^.

## Discussion

In the last century, the production of *B*. *thuringiensis* has been based on strains that express proteins related to sporulation (Cry and Cyt proteins) but with a low mass of spore and protein production. Even in a process with high yield such as fed-batch, because the focus is on the exponential growth phase, only the biomass is increased and not the crystal protein synthesis. Although biomass increases, the effects of cellular communication decrease protein synthesis during sporulation. An alternative approach has been to feed the culture during the stationary phase (sporulation) to redirect metabolism, achieving a slightly synthesis incresing^13,14^. Using this approach, in this work we show the mass production of a S-layer protein using the *B*. *thuringiensis* strain GP543. This is the first report of the production of a S-layer protein whose synthesis during the vegetative stage is associated with cell duplication. We also demonstrate that the crystalline inclusions constituted by this S-layer protein are toxic against the cattle tick *R*. *microplus*.

Although there are other proteins that are synthesized during the vegetative phase (VIP and SIP), these proteins are only virulence cofactors^15,16^ that are soluble and may be secreted but do not form crystalline protein inclusions. Similarly to most *B*. *thuringiensis* strains, GP543 showed a low conversion of glucose to biomass and barely reached X_max_ = 1.5 gDW · L^−1^ with Y_S/X_ = 0.17 g · g^−1^ at 10 g · L^−1^. Although *B*. *thuringiensis* regularly presents a low conversion of the carbon source into biomass, it is possible to modify the metabolic profiles in fed processes. These data suggest that carbon catabolite repression, influencing growth and perhaps protein synthesis is not an isolated case; most wild-type strains or modified strains have low oxidative metabolism and hence, many studies explore the effects of the culture media. We can see that culture media ranging from 10 to 40 g · L^−1^ of glucose (disregarding the yeast extract that can be used as a carbon source, varying from 3 to 10 g · L^−1^) results in low biomass, even applying a statistical experimental design or using response surface to the components of the culture medium. Efforts to improve metabolism through oxygenation have led to a small increase in biomass^17^ by maintaining a high oxygenation of at least 50%, which improves cellular metabolism and results in a slight increase in biomass of 2.4 g · L^−1^, although this increases the cost on a large scale. However, it is important to emphasize that high oxygenation during sporulation decreased toxicity, and when low oxygenation is applied during the sporulation phase, the toxicity increases considerably, although the investment in nutrients is very high at 30 g · L^−1^ of glucose and 12 g · L^−1^ yeast extract. These data suggest that many strains of *B*. *thuringiensis* may have a type of Crabtree metabolism, since at high concentrations of glucose, even in high oxygenation, they resulted in low values of biomass and product-forming fermentation by-products.

*B*. *thuringiensis* is widespread and can be isolated from soils and insect corpses, principally. In the insect haemolymph, *B*. *thuringiensis* is able to grow even with a low carbohydrates concentration, and thus metabolizing other carbon sources such as proteins. This metabolism is found in reports of alternative culture media in which fish or shrimp meat is used as substrate to growth *B*. *thuringiensis* strains, and even when the biomass obtained is low, the yields are very high with respect to the carbon source^18^. In other tests with several industrial by-products for the production of *B*. *thuringiensis*, one method is based on sugar cane molasses (50 g · L^−1^) as the carbon source and is supplemented with 50 g · L^−1^ of soybean flour as the nitrogen source. With this medium, a X_max_ = 3 gDW · L^−1^ with a yield of Y_S/X_ = 0.06 g · g^−1^ was obtained, without counting the soybean, to the advantage of 6%; the production of biomass was very poor, and moreover, this is typical for *B*. *thuringiensis*. In another report a culture medium was based on cheese whey supplemented with 15 g · L^−1^ sucrose as the carbon source, which reached a maximum biomass of X_max_ = 6 gDW · L^−1^ with a yield of Y_S/X = _0.3 g · g^−1^, thus doubling the biomass with three times less carbon source^19^. The cheese whey contains lactose from 4.5 to 5.3 g and β-D-galactopyranosyl, which does not cause inhibition by carbon catabolite repression and allows higher biomass to be produced. However, most of the proteins studied and produced by *B*. *thuringiensis* are related to the sporulation phase of the culture, where the nutrients have been exhausted and the only resources available are intracellular and divided between with the synthesis of the crystal and generation of the spore.

The cultures with a low glucose concentration are an alternative to regulate inhibition by carbon catabolite and have already been developed as feeding strategies that allow the biomass to reach up to Xmax = 72 g · L^−1^ in a fed-batch process^16^. Regardless of the increase in biomass, the strict relationship between the synthesis of toxic protein and sporulation has restricted the use of high-yield processes. In this report we identified by mass spectrometry analysis a S-layer protein from the GP543 strain whose production is associated with cell duplication. We calculated this parameter using the Luedeking and Piret (1959) relation by means of a logistic model of growth (see Material and Methods). To this end, we applied pressure to the kinetics of growth and the production and consumption of carbohydrates. The calculated values were used to solve the Luedeking and Piret equation, showing a coefficient of production associated with growth of α = 0.033 and a coefficient of production not associated with growth of β = 0.001. Since this is the first report of these values for a toxic protein produced by a *B*. *thuringiensis* strain, there are no comparisons of ranges. The GP543 strain has a high rate of protein synthesis during exponential growth and a small rate during the stationary phase. The rate of synthesis of the S-layer protein of the GP543 strain is directly related to the specific growth rate µ = 0.42 h^−1^; furthermore, α and β remained constant as the physiological age of the cells or the conditions of the process changed with R^2^ = 0.9. Perhaps the S-layer protein has an important structural function in the cell membrane, but GP543 strain forms protein crystal, with the yield Y_S/X_ = 0.14. Figure 3 shows that the protein concentration per gram of biomass remains constant during the exponential phase and increases slightly in the stationary phase since the synthesis of S-layer protein continues, even in the stationary phase, which agrees with the values α and β. The same tendency can be observed with the Western blot detection (Fig. 1) in which the synthesis is observed after the first hours of vegetative growth, but our data indicate that the S-layer protein is visible during all fermentation times. The relationship between production and cell growth shown by the GP543 strain makes it suitable for production in real processes of high yield, making it exceptional among the strains that are currently used for mass production.

*B*. *thuringiensis* strain GP543 was isolated from a *R*. *microplus* corpse and the combination of spores and crystalline inclusions that forms, showed toxicity against *R*. *microplus* engorged females in pathogenicity bioassays. The mix reached mortalities above 75% with 200 mg · mL^−1^ and above 60% with 100 mg · mL^−1^. Additionally, the S-layer protein caused a 13% inhibition of oviposition, this was an initial symptom low inhibition is an initial symptom of a drastic control effect because the oviposited eggs were infertile, since we show here that the lowest concentration of S-layer protein caused 85 to 92% inhibition of egg hatching. The purified protein S-layer also caused mortality on *R*. *microplus* engorged females at fourteen days in a range of 35.56% to 62.22% with concentrations between 10 to 200 mg/mL^−1^ and this demonstrate that the S-layer protein by itself is active against this thick. Thus, all the evidence here showed indicate that the strain GP543 of *B*. *thuringiensis* is potentially useful to be produced in enough amounts to constitute a viable alternative for the control of *R*. *microplus*.

## Methods

### Animal rights

The production of thick on cattle was approved by the Animal Experimentation and Ethics Committee of the Centro Nacional de Investigación Disciplinaria en Salud Animal e Inocuidad (CENIDSAI) part of the Instituto Nacional de Investigaciones Forestales Agricolas y Pecuarias (INIFAP). This research took ethic and methodological aspects into considerations in accordance with the Mexican regulations on use, housing and transportation of experimental animals (NOM-062-ZOO-1999 and NOM-051-ZOO-1995).

Animal care of the New Zealand White rabbit was at the Facultad de Ciencias Agropecuarias, Universidad Autonoma del Estado de Morelos, according the official Mexican regulations (NOM-062-ZOO-1999) in strict accordance with the Guide for the Care and Use of Laboratory Animals of the National Institute of Health (NIH), ensuring compliance with the stablished international regulations and guidelines. The production of the anti-S-layer antibody was approved by the Ethics Committee and Animal Experimentation of the Facultad de Ciencias Agropecuarias, Universidad Autonoma del Estado de Morelos.

### Microorganism and conditions of fermentation

The *B*. *thuringiensis* GP543 strain used in this research was kindly supplied by the Plant Parasitology Laboratory from the Center of Biological Research of the Autonomous University of Morelos State, Mexico. This wild-type strain was isolated from a female corpse of *R*. *microplus*. The inoculations for all experiments were carried out with a culture at a concentration of 5 × 10^6^ cells · mL^−1^. Modified HCT^20^ and GYS^21^ media were used in production. Fermentation conditions were carried out in a stirred-tank equipped with sensors and manual control systems for DO, pH, antifoam, impeller speed, aeration rate and temperature. The fermentor dimensions are as follows: 7 L of total volume, 5 L of working volume, and one impeller Rushton turbine (Applikon controller P140). The fermentor was inoculated with GP543 strain at 5% (v/v) with the pre-culture. The fermentor was kept at 1 vvm, pH = 7, and 30 °C ± 1. Samples were taken every 2 hours.

### Estimation of biomass yield and cell productivity

The substrate to biomass conversion was calculated as the yield (Y_S/X_ = ΔX/ΔS; X = gDW · L^−1^, S = g · L^−1^), the biomass X_max_ corresponded to the maximum cell concentration, t_max_ = h, the total protein concentration was quantified by the Bradford technique^22^ as P = mg · mL^−1^, and cell productivity was calculated as the yield Y_X/P_ = ΔP/ΔX. The volumetric productivity was calculated as Qp = P/t, where (t) is the total time of fermentation.

### Anti-S-layer antibody production

A New Zealand White rabbit was immunized with the solubilized and purified by anion-exchange chromatography S-layer protein produced by the GP543 strain by subcutaneous injections. It was injected three times at five sites on the back of the rabbit every 15 days. The dosis were 1 mg of protein mixed with incomplete Freund’s adjuvant. Five days after the third immunization the rabbit was bled. The blood was allowed to stand for one hour at room temperature and centrifuged five minutes at 2000 rpm to recovered and store the serum. Detection was done with anti-GP543 SL polyclonal antibody (dilution, 1:30,000; 1 h) and visualized with a goat anti-rabbit antibody coupled with horseradish peroxidase (Sta. Cruz Biotechnology) (dilution, 1:50,000; 1 h), followed by Super Signal chemiluminescent substrate (Thermo Scientific) as described by the manufacturers^5^.

### Western blot analysis

From a fermentation batch, 1 mL of sample was taken every 2 hours. The samples were centrifuged at 12,000 × g for 10 minutes, the supernatant was discarded, the pellet was suspended in 100 µL of phosphate buffer and 1 µL of PMSF was added to the new sample. From the concentrated sample, 6 µL were taken, mixed with Laemmli buffer and solved by SDS-PAGE in 8% gels. The full set of samples was solved in duplicate, with one of the gels stained with Coomassie blue and the other transferred to PVDF membrane (BioRad; Alfred Nobel Drive Hercules, California 94547). For the Western blot, the membrane was blocked with 2% BSA in phosphate buffer plus 0.1% Tween 20 (PBS-T) for 2 hours at room temperature. The detection was achieved with the primary anti-S-layer antibody at a concentration of 1:30,000 in PBS-T for 1 hour followed by the secondary anti-rabbit antibody coupled to peroxidase (Sta. Cruz Biotechnology) at a concentration of 1:50, 000 in PBS-T for 1 hour. The membrane was developed using the substrate Supersignal West pico (Thermo Scientific).

### Logistic growth model

A simple logistic model was employed to describe cell growth to accurately determine the specific growth rate, which was used to replace the model for the production and consumption of substrate by the following equation:

1 $$\frac{\partial X}{\partial t}=\mu \,{\rm{\max }}\,X\,(1-\frac{X}{X\,{\rm{\max }}})$$

### Logistic model for production

The production kinetics were evaluated by the Luedeking-Piret equation^23^, where α is a constant of production associated with growth and β is a constant not associated with growth:

2 $$\frac{{\rm{d}}p}{{\rm{d}}t}=\alpha \frac{{\rm{d}}N}{{\rm{d}}t}+\beta N$$

The logistic growth model (Xt) was replaced in the Luedeking-Piret equation and then integrated:

3 $$Pt={P}_{o}+\,\alpha Xo\,[\frac{{e}^{\mu t}}{1-[(\frac{{X}_{o}}{X\,{\max }})(1-{e}^{\mu t})]}]+\beta \frac{X\,{\max }}{\mu }\,\mathrm{ln}\,[1-(\frac{{X}_{o}}{X\,{\max }})(1-{e}^{\mu t})]$$

### Logistic model for consumption of substrate

The consumption kinetics of glucose were evaluated by a modified Luedeking-Piret equation in which three growth variables, substrate consumption, biomass increase and production, were evaluated, as shown in the following equation:

4 $${S}_{o}-St=(\frac{1}{{Y}_{S/X}}+\frac{\alpha }{{Y}_{S/p}})(X-{X}_{o})+(\frac{\beta }{{Y}_{S/p}}+Ke)\frac{X\,{\max }}{\mu }\{\mathrm{ln}\,[1-(\frac{{X}_{o}}{X\,{\max }})(1-{e}^{\mu t})]\}$$

### Immersion bioassays

Pathogenicity bioassays were carried out with the GP543 strain against engorged ticks by immersion^24^. Increasing concentrations of spore-protein complex ranging from 50 to 300 µg · mL^−1^ were employed and distilled water was used as a negative control. The female ticks were placed individually into one well (Cell Wells NUNC) of 24 wells and remained in immersion for five minutes in 1 mL of spore-protein solution. The ticks were dried and placed in a clean well and incubated for 14 days in a humid chamber at 90% RH and 25 ± 2 °C. For pathogenicity bioassay using pure S-protein, the concentrations ranged from 10 to 200 µg · mL^−1^. A sample of 1200 engorged ticks was homogenized by weight and divided in experimental units. To each experimental unit a concentration of purified S-layer protein was applied.

### Purification of the crystal inclusion of s-layer protein produced by the GP543 strain

The bacteria was grown in petri dishes containing solid HCT^20^ medium. The spore-crystal mixture was collected in 5 ml of sterile water and centrifuged for 10 min at 10,000 rpm. The pellet containing only spores was discarded and the supernatant was recovered. The process was repeated for five times until the spores were removed. The last supernatant was centrifuged at 19,000 rpm for 30 min to recover the crystal inclusions. The crystals were solubilized in 50 mM Na_2_CO_3_, pH 10.5, with 0.2% β-mercaptoethanol and purified by anion-exchange chromatography in a Q-Sepharose column (HiTram^TM^), using liquid chromatography^5^.

### Experimental design and analysis

Each experimental unit had 24 *R*. *microplus* individuals, and each treatment was used in each experimental group. The ticks were homogenized by weight; each individual was weighed using an analytical balance (OHAUS AS 120; OHAUS Corporation). With these weights, we generated a graph (frequency *vs* weight) with a Gaussian distribution, which allowed us to discard the ends of the distribution (heaviest and lightest) to select a homogeneous sample. After this selection, the ticks were distributed completely at random in Cell Wells NUNC. This experiment was replicated four times, and the mortality percentages were calculated at 8, 10, 12 and 14 days after treatment. A completely randomized experimental design was employed to evaluate the range of concentrations of the spore-protein complex and analysed by Tukey’s multiple range tests. SAS software (Version 9.0) was employed for statistical analyses.

### Mortality percentage of *R*. *microplus*

Dead and live ticks were counted at 8, 10, 12 and 14 days after the exposure to treatment. It was determined by observation using a stereoscopic microscope that the ticks were dead when they did not have abdominal movement even with the application of a tactile stimulation with a brush and they remained motionless until the end of the experiment.

### Percentage inhibition of oviposition

The percentage of oviposition (% I.O.) was determined^24^ after 14 days of incubation. The eggs laid throughout the experiments were collected and weighed by experimental unit using an analytical balance (OHAUS AS 120; OHAUS Corporation). The equation described was used to determine % I.O.:

5 $$ \% {\rm{I}}.\,{\rm{O}}.=({{\rm{PQL}}}_{{\rm{t}}}/{\rm{PQLT}}-{{\rm{PHL}}}_{{\rm{t}}}/{\rm{PHLT}})\ast 100$$

where PQL_t_ = weight of female ticks in the experimental unit, PQLT = weight of female ticks in the control unit, PHL_t_ = weight of oviposited eggs in the experimental unit and PHL_T_ = weight of oviposited eggs in the control stock.

### Protein sequencing

The solubilized protein from crystal-spore was solved by 9% SDS-PAGE. The band of around 100 kDa was excised from the gel, trypsin digested^25^ and analyzed with an Ultraflex Extreme MALDI-TOF/TOF (Bruker). Peptide mass fingerprinting analyses were performed using the Flex Analysis 3.5 software (Bruker) and the Mascot Server (Matrix Science) using the NCBI database. Accepted peptide scores were equal or higher than 95% identification confidence threshold^23,26^.

## Supplementary information

Mass production of a S-layer protein of Bacillus thuringiensis and its toxicity to the cattle tick Rhipicephalus microplus.

## Data Availability

The datasets generated during and/or analyzed during the current study are available from the corresponding author on reasonable request.
