# Supplementary material for: Mass production of a S-layer protein of Bacillus thuringiensis and its toxicity to the cattle tick Rhipicephalus microplus
Source: Sci Rep. 2019 Nov 26;9:17586. doi: 10.1038/s41598-019-53854-3 (PMC6879537; doi:10.1038/s41598-019-53854-3)
Supplement: Supplementary file 1 — Mass production of a S-layer protein of Bacillus thuringiensis and its toxicity to the cattle tick Rhipicephalus microplus. [file 41598_2019_53854_MOESM1_ESM.docx]

Supplementary information

Mass production of a S-layer protein of *Bacillus thuringiensis* and its toxicity to the cattle tick *Rhipicephalus microplus*.

Caleb Lormendez C., Manuel Fernandez-Ruvalcaba, Markis Adames-Mancebo, Victor Manuel Hernandez-Velazquez, Fernando Zuñiga-Navarrete, Gabriela Flores-Ramirez, Laura Lina-García, Guadalupe Peña-Chora

Tabla 1. Partial sequence of the S-layer protein that is toxic to *R*. *microplus*. Protein sequence coverage 58%.

|  | Organism | |
| --- | --- | --- |
|  | *Bacillus thuringiensis* GP1 (AAY28601.1) | *Bacillus cereus* SJ1  (EFI64127.1) |
| Peptide | Homology |  |
| SFPDVPAGHWGLDSINYLVDK | 100 | 100 |
| GAIEGKPDGTYAPAEEIDR | 100 | 100 |
| GAIEGKPDGTYAPAEEIDRASAAK | 100 | 100 |
| IMAITLGLKVEEGAQPSFK | 100 | 100 |
| ASFASMIVGAYNLK | 100 | 100 |
| VNGELVTKFEDLLDHWGEEK | 100 | 100 |
| FEDLLDHWGEEK | 100 | 100 |
| ANILIHLGLSEGTGGNK | 100 | 100 |
| ANILIHLGLSEGTGGNKWEPNK | 100 | 100 |
| LAAEDVTLEGDK | 100 | 83.33 |
| LAAEDVTLEGDKAVAIEASADGTSAVVTLG | 100 | 93.33 |
| LTFDDDR | 100 | 100 |
| LTFDDDRAGQAVAFK | 100 | 93.33 |
| LNDEKGNADVEYLNLADHDVK | 100 | 95.24 |
| GNADVEYLNLADHDVK | 100 | 93.75 |
| FVANNLDGSSANIFEGGVATSTTGK | 100 | 92.0 |
| LAVGIKPADYKVEVQVTK | 100 | 88.89 |
| RGGLTVSNTGIITVK | 100 | 90.0 |
| NAVFALDADNDGVVNYGSK | 100 | 94.74 |
| LSGKDFALNSQNLVVGEK | 100 | 100 |
| LVATIAGEDKVVDPGSISIK | 100 | 100 |
| SSNHGIISVVNNYITAEAAGEATLTIK | 100 | 100 |
| VTTDSR | 100 | 100 |
| VGQYGASPDTKLDLNVSDTVAYQLSK | 100 | 88.46 |
| YTSDRVYSDPENLEGYAVESK | 100 | 95.24 |
| VYSDPENLEGYAVESKNEK | 100 | 94.44 |
| ATIEIVQETIAIK | 100 | 100 |
| SVNFKPVQTENFVEK | 100 | 100 |
| SVNFKPVQTENFVEKK | 100 | 100 |
| INIGTVLELEKSNLDDIVK | 100 | 100 |
| SGDEQGKLYLDR | 100 | 91.67 |
| LYLDR | 100 | 100 |
| NGDAVFNAGDVNLGYVTVSQTSDSALPNFK | 100 | 93.33 |
| VLGEKDVLTSEIGSQAVHVNVLNNPNL | 100 | 88.89 |
